# Assessment of Selected Chemical and Morphological Properties of *Lonicera* var. *kamtschatica* and *Lonicera* var. *emphyllocalyx* Treated with Gaseous Ozone

**DOI:** 10.3390/molecules29153616

**Published:** 2024-07-31

**Authors:** Oskar Basara, Józef Gorzelany

**Affiliations:** 1 Department of Food and Agriculture Production Engineering, University of Rzeszów, St. Zelwerowicza 4, 35-601 Rzeszow, Poland; oskarb@dokt.ur.edu.pl; 2 Doctoral School, University of Rzeszów, St. Rejtana 16C, 35-959 Rzeszow, Poland

**Keywords:** *Lonicera caerulea* L., ozonation, ascorbic acid, polyphenol content, antioxidant value, morphological properties

## Abstract

*Lonicera caerulea* L. fruits are a rich source of vitamins, organic acids, and phenolic compounds, which are characterised by their health-promoting properties. The content of bioactive compounds in this fruit may vary depending on the cultivar and the harvest date. This study analysed the effect of applying 5 ppm gaseous ozone for 1, 3, and 5 min on the chemical properties of *L. kamtschatica* varieties and newly created clones of *L. emphyllocalyx* for three years of cultivation. The fruits harvested from *L. emphyllocalyx*, depending on the year of harvest, had significantly larger size and weight compared to *L. kamtschatica*. On average, the acidity of the *L. emphyllocalyx* clones was 6% higher than other tested varieties. The average content of ascorbic acid was highest in *L. emphyllocalyx* clone ‘21-17’—57.80 mg·100 g^−1^; the year of harvest will significantly affect the content of vitamin C, reaching the highest level in 2022—53.92 mg·100 g^−1^. The total content of polyphenols was significantly dependent on the year of cultivation; reaching, on average, 54.8% more in 2022 compared to the rest of the years. The total antioxidant value using the FRAP, DPPH, and ABTS methods varied depending on the variety; exposure to ozone significantly increased the antioxidant value in each case. On the basis of the study, both botanical varieties can be used in food processing. Gaseous ozone exposure can significantly influence chemical composition, increasing the health-promoting value of fruit.

## 1. Introduction

*Lonicera caerulea* L. is a known species found in forests and mountainous and low-lying wet regions of Europe, North Asia, and North America. The genus *Lonicera* consists of over 200 cultivars; the most commonly planted are blue honeysuckle, originating in Russia, Japan, and Canada [1]. *Lonicera caerulea* L. has several botanical varieties that come from Russia: *L. caerulea* var. *edulis*, *L. caerulea* var. *altaica*, *L. caerulea* var. *boczkarnikovae*, and *L. caerulea* var. *kamtschatica*; and from the Japanese island of Hokkaido: *L. caerulea* var. *emphyllocalyx* [2]. *L. kamtschatica*, commonly called the ‘kamczatka berry’ in Poland, is a well-known botanical variety of *Lonicera caerulea* L., recognised for its cylindrical shape berries, which are among the earliest ripening fruit plants in Poland [3]. *L. emphyllocalyx*, also known as the ‘haskap berry’, is a lesser-known variety characterised by fruit shape more reminiscent of highbush blueberries, lesser fruit falling, and later ripening time compared to *L. kamtschatica* [4].

*L. caerulea* L. fruits are primarily composed of fibre, protein, calcium, and magnesium, and they also have high concentrations of glucose and fructose, with traces of sucrose and sorbitol. They are a rich source of polyunsaturated fatty acids, especially linoleic acid and are notable for their high ascorbic acid content [5]. Studies have showed the richness of *L. caerulea* L. in anthocyanin compounds, with cyanidin 3-glucoside being the predominant anthocyanin, comprising 79–92% of the total content. Other anthocyanins present in smaller amounts include cyanidin 3,5-diglucoside (4.27%), cyanidin 3-rutinoside (2.07%), peonidin 3-glucoside (3.44%), and pelargonidin 3-glucoside (0.83%) [6]. *L. caerulea* berries are characterised by high content of polyphenols ranging from 426.1 to 622.52 mg GAE·100 g^−1^ f.w., which have strong antioxidant properties: 68.68–89.62% (DPPH inhibiton), 1.91–2.26 mM TE·100 g^−1^ f.w. (ABTS), and 27.96–49.90 µM Fe^2+^·g^−1^ f.w. (FRAP) [7,8]. Berries are a known source of vitamin C (ascorbic acid), ranging from 30.8 to 62.6 mg·100 g^−1^ f.w. [8]. The berries are used to produce spreads, juices, and wines, and the fruits are also dried to extend their shelf life and change the content of health-promoting compounds [9]. Due to their exceptional richness in anthocyanin compounds, these fruits can be regarded as excellent sources of natural colourants, offering a range of colours from red to purple, pink, or blue. They have potential applications in various sectors, including food, pharmaceuticals, and cosmetics.

Ozone (O_3_) is a powerful oxidising agent commonly used in its gaseous form, without leaving any trace of toxic residues. Ozonation is an environmentally friendly, non-thermal food preservation method that does not decrease the quality of the fruit [10]. If used in the right dose, it can change metabolic processes, increasing a total amount of bioactive compounds and enhancing antioxidant value. The profile of phenolic compounds and the quantity of compounds may change when ozone gas is used [11]. The gaseous ozonation positively impacts fruit storage by reducing water loss, microbial load, and decreasing ethylene released from the treated fruit, thus extending the shelf-life of produce [12,13]. Food processing faces the challenge of preserving bioactive compounds in food while ensuring a long shelf life and maintaining fresh-like sensory quality. The processing of fruits can alter the sensory quality and levels of polyphenols; water loss caused by processing methods, such as heating, might change the concentration of some compounds. Ozone interacts with certain organic compounds in food, leading to the formation of by-products such as ketones, carboxylic acids, and aldehydes, which do not pose any risk to human health [13,14]. Ozonation may be superior to thermal processing for food preservation, as it minimises the loss of nutrients during storage and can increase amounts of phenolic compounds and ascorbic acid [15].

The purpose of our study was to compare the chemical compositions of the berries of *L. caerulea* var. *kamtschatica* (‘Vostorg’, ‘Jugana’, and ‘Aurora’ cultivars) and *L. caerulea* var. *emphyllocalyx* (‘Lori’ cultivar and ‘21-17’, ‘139-24’ clones) and the potential use of the *L. emphyllocalyx* fruit and ozone fumigation in food processing. Various studies must be conducted before new breeding clones of *L. caerulea* are registered and introduced into cultivation. The results of studies on the chemical properties of *L. emphylocallyx* clones ‘Lori’, ‘21-17’ and ‘139-24’ have not yet been published. The effect of ozone fumigation on the antioxidant activity, and total amount of polyphenolics and ascorbic acid was also examined.

## 2. Results and Discussion

### 2.1. Changes in pH and Acidity in L. kamtschatica and L. emphyllocalyx Fruits in Relation to Ozonation Time

**Figure 1:**
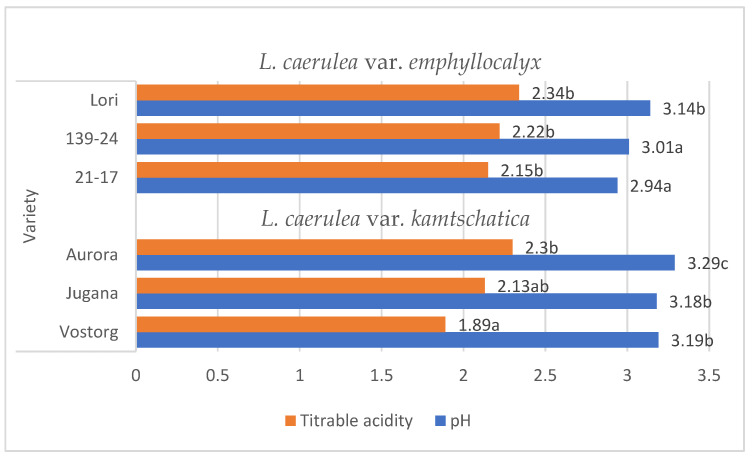
Average pH and total acidity (g·100 g^−1^) of *L. kamtschatica* and *L. emphyllocalyx* fruits obtained. Data are expressed as mean values (*n =* 3) ± SD; SD: standard deviation. Mean values with different letters are significantly different (*p* < 0.05).

*Lonicera caerulea* L. berries are rich source of organic acids (e.g., citric, shikimic, quinic, tartaric acid), the contents of individual organic acids significantly influence the taste qualities of ripe fruit and their acceptability to consumers. A characteristic of *Lonicera caerulea* L. berries is their high level of acidity, which has a very negative impact on the sensory experience [15]. The organic acid content of the fruit decreases with successive stages of ripening. These organic acids are degradable and can change in concentration due to various factors, such as temperature [9,16,17]. The average pH values of the *L. kamtschatica* and *L. emphyllocalyx* berries were 3.18–3.29 and 2.94–3.14, respectively [Figure 1].

**Figure 2:**
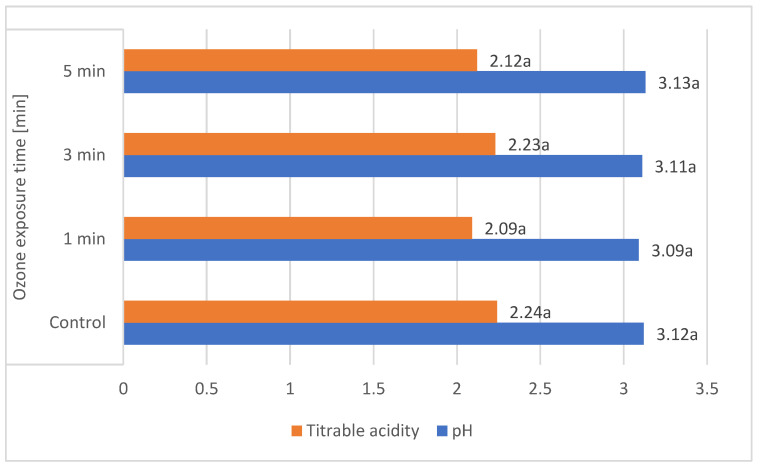
Average pH and total acidity (g·100 g^−1^) of *L. kamtschatica* and *L. emphyllocalyx* fruits subjected to gaseous ozone exposure. Data are expressed as mean values (*n =* 3) ± SD; SD: standard deviation. Mean values with different letters are significantly different (*p* < 0.05).

These results are comparable to those obtained by other authors. According to Gerbrandt et al., 2018 [18], the fruit pH of *L. caerulea* ranged from 2.42 to 3.57, depending on the cultivar and location. According to MacKenzie et al., 2018 [19], the pH of *L. caerulea* berries ranged from 3.0 to 3.4, depending on cultivar and harvesting year. In this study, *L. emphyllocalyx* was characterised by a significant lower-than-6% average pH compared to analysed varieties of *L. kamtschatica*. The use of ozone gas did not significantly affect the pH. of the fruit [Figure 2].

**Figure 3:**
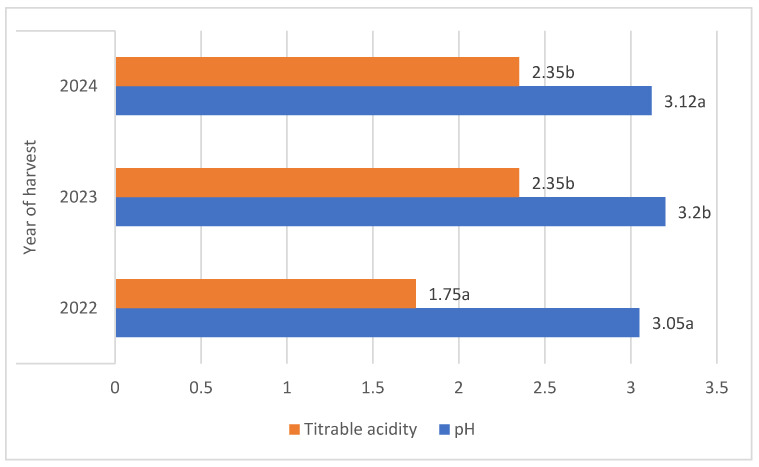
Average pH and total acidity (g·100 g^−1^) of *L. kamtschatica* and *L. emphyllocalyx* fruits depending on year of harvest. Data are expressed as mean values (*n =* 3) ± SD; SD: standard deviation. Mean values with different letters are significantly different (*p* < 0.05).

There were significant differences in the fruit harvest date; berries of both botanical varieties harvested in 2023 were characterised by an average pH that was 5% higher compared to other years [Figure 3].

The pH value is varied compared to other fruit species popular in Poland, e.g., highbush blueberry fruit: 2.76–3.33 [20], raspberry: 3.72 [21], strawberry: 3.20–4.00 [22].

The average titrable acidity values of the *L. kamtschatica* and *L. emphyllocalyx* berries were 1.89–2.30 g·100 g^−1^ and 2.15–2.34 g·100 g^−1^, respectively [Table 1]. The results obtained in this study are comparable to those obtained by Gerbrandt et al., 2018 [18]; the titrable acidity of *Lonicera caerulea* L. berries were from 1.64 to 3.52% CAE. In this study, *L. kamtschatica* was characterised by a significant lower-than-6% average titratable acidity in comparison to *L. emphyllocalyx*. There were no significant changes in total acidity after the use of ozonation. Berries of all tested varieties harvested in year 2022 were characterised by a significant lower-than-25% titrable acidity in comparison to other years. A decrease in the pH and an increase in the acidity of the fruit can significantly affect the organoleptic properties of the fruit, making it more or less desirable to consumers [23]. The significantly higher pH and lower acidity of berries may be related to the content of bioactive compounds such as polyphenols or ascorbic acid [24]. Berries of *L. kamtschatica* are characterised by a higher pH and lower acidity average, making them potentially better in taste. In our study, the year of harvest had a significant impact on the pH and acidity of *L. kamtschatica* and *L. emphyllocalyx*, which coincides with the results obtained in the study of MacKenzie et al., 2018 [19], where there were significant differences between the year of harvest. Obtained results are comparable with other popular berries grown in Poland, e.g., cranberry (1.56–1.60 g·100 g^−1^) [25], raspberry (1.67–1.76 g·100 g^−1^) [21], red currant (0.7–1.6 g·100 g^−1^) [26].

### 2.2. Contents of Bioactive Compounds in L. kamtschatica and L. emphyllocalyx Berries

Ascorbic acid, known as vitamin C, is a water-soluble vitamin and antioxidant essential for human health. It plays a vital role in the synthesis of collagen, the absorption of iron, the maintenance of the immune system, and the repair of tissues. Ascorbic acid also helps protect cells from damage by free radicals [27,28]. The average content of ascorbic acid in *L. kamtschatica* and *L. emphyllocalyx* was 49.74–54.32 mg·100 g^−1^ and 53.13–57.80 mg·100 g^−1^ [Table 1].

**Table 1:** Average contents of ascorbic acid, total polyphenols content, and antioxidant value of ozonated *L. kamtschatica* and *L. emphyllocalyx* fruits obtained in three harvesting years.

Variable		Ascorbic Acid [mg·100 g^−1^]	Total Polyphenols Content [mg·100 g^−1^]	FRAP [µM Fe^2+^·g^−1^]	DPPH [% Inhibition]	ABTS [µM TE·g^−1^]
Variety		*L. caerulea* var. *kamtschatica*				
	Vostorg	51.05 ab	310.12 d	28.97 c	82.16 ab	0.33 c
	Jugana	49.74 a	261.38 ab	29.81 b	79.89 a	0.31 a
	Aurora	54.32 d	278.08 c	25.42 a	79.50 a	0.36 d
		*L. caerulea* var. *emphyllocalyx*				
	21-17	57.80 bc	276.05 bc	29.53 bc	83.10 ab	0.35 c
	139-24	53.13 cd	231.59 a	28.00 b	82.87 ab	0.34 b
	Lori	54.08 d	286.92 b	29.48 c	85.41 b	0.36 c
SL		***	***	***	***	***
Ozone exposure time [min]	Control	51.74 a	251.57 a	27.78 a	82.37 cd	0.33 a
	1 min	53.98 a	266.78 b	28.22 ab	81.62 ab	0.35 b
	3 min	51.69 a	299.71 c	28.51 ab	85.14 d	0.34 ab
	5 min	52.81 a	278.27 b	29.64 b	79.15 a	0.35 b
SL		ns	***	***	***	***
Year of harvest	2022	53.92 b	390.88 b	32.69 b	83.44 b	0.33 a
	2023	50.43 a	212.31 a	22.14 a	85.50 b	0.35 b
	2024	53.18 b	219.05 a	30.78 b	79.48 a	0.35 b
SL		***	***	***	***	***

Data are expressed as mean values (*n =* 3) ± SD; SD: standard deviation. Mean values with different letters are significantly different (*p* < 0.05). SL: level of significance; ns: not significant; ***: *p* < 0.001.

The results obtained in this study are comparable with those obtained by Ochmian et al., 2008 [29]. Researchers have shown that the content of ascorbic acid varies depending on the variety and ranges from 40.5 to 98.0 mg·100 g^−1^. According to Ćesonie et al., 2021 [30], the content of ascorbic acid in *L. caerulea* varies and ranges from 14.55 to 53.58 mg·100 g^−1^ depending on the cultivar. The average content of ascorbic acid was higher in *L. emphyllocalyx* fruits, only the ‘Aurora’ variety *L. kamtschatica* had a comparable amount of vitamin C—54.32 mg·100 g^−1^. On average, *L. kamtschatica* varieties had 6.1% less ascorbic acid in comparison to tested *L. emphyllocalyx* varieties. The use of ozone did not significantly affect the ascorbic acid content. In the year 2023, significantly less vitamin C was recorded in fruit of *L. kamtschatica* and *L. emphyllocalyx* compared to other years. In 2023, there was, on average, 5.9% less ascorbic acid compared to other years of cultivation. According to Ochmian et al., 2008 [29], the content of ascorbic acid varied depending on the variety and year of harvest, which coincides with the results obtained in this experiment. Most varieties of *L. emphyllocalyx* are characterised by the higher average content of vitamin C compared to *L. kamtschatica*, thus increasing their health-promoting properties. The vitamin C content in the fruit is much higher compared to other species grown in Poland, e.g., blackthorn, 21.94 mg·100 g^−1^; blackberry, 33.85 mg·100 g^−1^ [31]; strawberry, 23.8–45.17 mg·100 g^−1^ [32]; raspberry, 12.10–45.52 mg·100 g^−1^ [33].

The phenolic compounds, primarily anthocyanins, in *L. caerulea* fruit extract demonstrate anti-inflammatory effects. They reduce cellular damage under oxidative stress in in vitro cultures of rat microsomes and decrease ROS production in cultures of proinflammatory gingival fibroblasts [34,35]. The average total polyphenol content value in *L. kamtschatica* and *L. emphyllocalyx* ranged from 261.38 to 310.12 mg·100 g^−1^ and 231.59 to 286.92 mg·100 g^−1^. Results obtained in this study are comparable with those obtained by Pažereckaitė et al., 2020 [36], where the total polyphenol content ranged from 282 to 781 mg·100 g^−1^, depending on the tested cultivar. The choice of variety had a significant impact on the total content of phenolic compounds; the average content of compounds was higher in *L. kamtschatica*. The average content of polyphenols in the tested *L. kamtschatica* varieties was lower by 6.5% compared to the *L. emphyllocalyx* varieties. According to Kucharska et al., 2017 [3], different varieties contained different profiles of phenolic compounds; some varieties contained compounds that other varieties did not, e.g., variety ‘Vostorg’ did not contain di CQA 2 and di CQA 3, compared to other studied varieties. A different profile of phenolic compounds may significantly affect the total polyphenol content. Fruits with a larger diameter harvested at the optimal harvest time may contain higher anthocyanin content due to the larger skin area [9]. There was a significant effect of exposure to ozone gas (ozonation for 3 min); it significantly increased the content of phenolic compounds in fruits. The average total amount of phenolic compounds was higher by 11.5% in varieties subjected to ozone exposure of 5 ppm·3 min. The results obtained in this experiment coincide with those obtained by Piechowiak et al., 2019 [14], where ozonated fruits had a higher content of phenolic compounds. Significant differences in the total polyphenol content in individual years of cultivation prove the importance of cultivation conditions. The total amount of phenolic content in *L. kamtschatica* and *L. emphyllocalyx* fruits was higher by 54.8% in 2022. The total polyphenol content in fruits of *L. kamtschatica* and *L. emphyllocalyx* are comparable to other popular berries grown in Poland, e.g., strawberries—238.0 mg GAE·100 g^−1^ f.w. [22], highbush blueberry—424.72 mg GAE·100 g^−1^ f.w. [37], and blackberry—247.25 mg GAE·100 g^−1^ f.w. [31].

Among bioactive compounds, polyphenols are the most abundant group of chemical compounds found in *L. caerulea* fruits. Fruit extract containing phenolic compounds is a highly effective antioxidant agent, significantly reducing reactive oxygen species (ROS) produced by immune cells during inflammation [38]. Antioxidant activity of berries primarily depends on their chemical composition, particularly the content and varied structure of polyphenolic compounds, which influence their antioxidant potential [38]. The average FRAP antioxidant activity is similar in all tested varieties, ranging from 25.42 to 28.97 µM Fe^+2^·g^−1^ in *L. kamtschatica* berries and 28.00 to 29.53 µM Fe^+2^·g^−1^ in fruits of *L. emphyllocalyx*. The results obtained in this study are comparable to those obtained by Rupasinghe et al., 2012 [7], where FRAP antioxidant activity was dependent on the tested variety and was within the range from 27.96 to 46.90 µM Fe^+2^·g^−1^. The average DPPH antioxidant activity in *L. kamtschatica* and *L. emphyllocalyx* was 79.50–82.16% and 82.87–85.41%, respectively. In the study by Lee et al., 2018 [39], the researchers obtained results similar to this experiment. The DPPH value ranged from 33.9 to 95.96%, depending on the concentration of the extract. The antioxidant value of ABTS varied; in the case of *L. kamtschatica,* it was in the range of 0.31–0.36 mM TE·g^−1^, and in the case of *L. emphyllocalyx*, it was in the range of 0.34–0.36 mM TE·g^−1^. The results obtained in this study are comparable to those obtained by Oszmiański et al., 2016 [40], where the antioxidant value of ABTS was 0.30 mM TE·g^−1^. The tested *L. emphyllocalyx* varieties showed, on average, higher antioxidant values tested using the FRAP, DPPH, and ABTS methods. Our previous study [8], as well as other studies [30,41], indicate the possibility of differences in the antioxidant value of different botanical varieties but also varieties of the same species. The results obtained in this experiment indicate that *L. emphyllocalyx* has more effective antioxidant activity, thus more significantly reducing ROS compared to the tested variety of *L. kamtschatica*. The effect of exposure to ozone gas was varied; however, the antioxidant value in ozonated fruits was higher compared to the control sample. In our previous study [26], the effect of ozonation within one variety was different depending on the chosen method of measuring the antioxidant value. Fruits exposed to ozone showed higher antioxidant content due to a lower loss of polyphenols compared to control samples. Climatic conditions and the date of harvest in particular years may significantly affect the saturation of the fruit with bioactive compounds, including the antioxidant value of berries [41]. In 2023, in the months during which the fruit ripened (May and April), the lowest average monthly temperature was observed during the years studied, which could have a significant impact on the content of health-promoting compounds in the fruit. The berries of *L. kamtschatica* and *L. emphyllocalyx* in 2023 were characterised by the lowest average content of bioactive compounds. This indicates the influence of monthly average temperature on vegetation, which may be directly related to the origin of plants from cold regions of the world. Research on the antioxidant activity of *L. caerulea* extracts has shown that the fruits of this plant possess strong antioxidant properties. Given the link between modern diseases and long-term oxidative stress, these strong antioxidant properties suggest that *L. caerulea* may be important not only for disease prevention but also for the treatment of various conditions.

### 2.3. Determination of the Morphology and Colour of Berries

**Figure 4:**
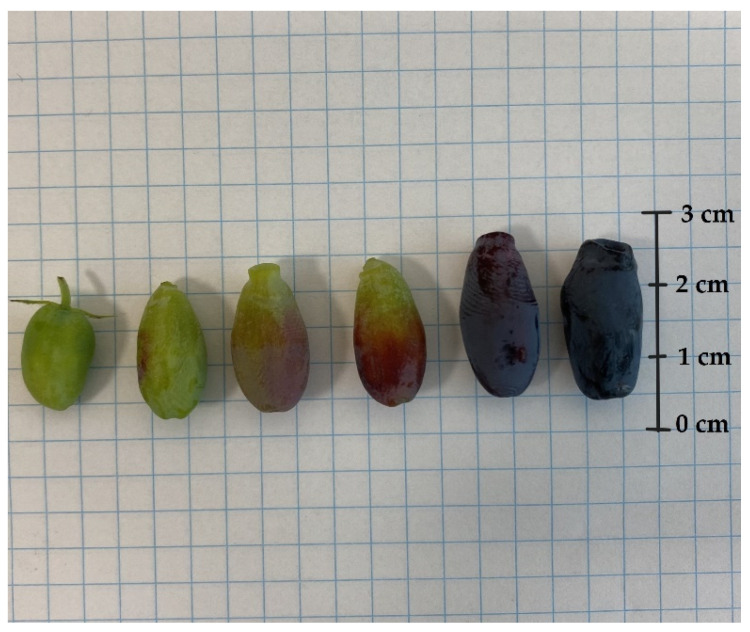
*L. caerulea* var. *kamtschatica* berries at different stages of maturity.

**Figure 5:**
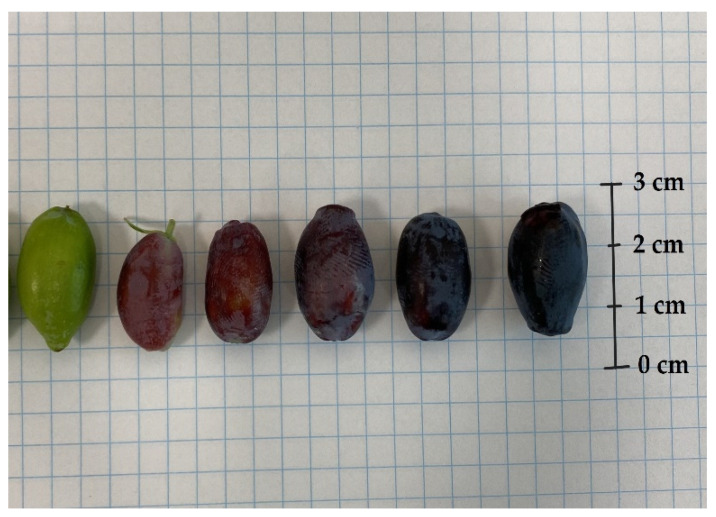
*L. caerulea* var. *emphyllocalyx* berries at different stages of maturity.

In each year of the experiment, fruits from all cultivars were harvested six or seven times. The mass, size, and colour of the berries are crucial factors in assessing their quality and appeal to customers. Significant differences between the fruits of *L. kamtschatica* and *L. emphyllocalyx* were found [Figure 4 and Figure 5].

The average length and width of berries were 21.49 and 8.83 cm for *L. kamtschatica* and 20.09 and 10.70 cm for *L. emphyllocalyx* [Table 2].

A significant difference in fruit size was observed. The shape of *L. kamtschatica* fruits was more cylindrical, the average fruit length was longer, and the width was smaller, compared to *L. emphyllocalyx* fruits. The fruits of *L. emphyllocalyx* were characterised by a rounder shape, slightly resembling a highbush blueberry. On average, the weight of *L. kamtschatica* was lower, 35.5%. Larger fruits may be more attractive to a potential consumer. The *L. emphyllocalyx* varieties were characterised by fruits with a significantly average darker colour, 28.30, with a lower average redness value, 3.01. The shape and weight of the fruit have a significant impact on the transport and storage process.

## 3. Materials and Methods

### 3.1. Material

The fruits of the *L. kamtschatica* cultivars ‘Vostorg’, ‘Jugana’, and ‘Aurora’ and *L*. *emphyllocalyx* ‘Lori’, ‘21-17’, and ‘139-24’ were obtained from a nursery crop located in Tyczyn (49°57′52″ N, 22°2′47″ E, Podkarpackie Voivodship, Poland) in the years 2022–2024. Both species were grown in pots filled with substrate-containing peat, sand, and perlite in a ratio of 20:1:1. In 2022, 3–4 m of Osmocote Exact (ICL, Sydney, Australia) and a 2.0 kg·m^−3^ concentration of substrate were added. In 2023–2024, the plants were periodically supplied with Kristalon Blue (Yara, Oslo, Norway) in a concentration of 0.4 kg·m^−3^. The average monthly temperature and rainfall from 2022–2024 in the period of March to June in Tyczyn are represented in Table 3.

The fruits of the analysed cultivars were harvested by hand at the stage of their maturity, 1000 g each. The harvest time varied significantly, depending on the tested variety [Table 4]. In 2024, due to weather conditions and rapid plant development, fruit ripening and harvesting took place approximately 2 weeks earlier, compared to previous years. Immediately after harvest, the fruits were subjected to chemical analysis.

### 3.2. Ozone Treatment of Berries

Before harvest, the whole *L. kamtschatica* and *L. emphyllocalyx* plants with fruits were subjected to ozonation treatment. The plants were randomised into four batches containing 10 plants, each bearing around 250 g of fruits. Gaseous ozone was used at a concentration of 5 ppm for 1 min, 3 min, and 5 min (flow 40 g O_3_·h^−1^, temperature 20 °C). The ozone was produced with a KORONA A 40 Standard (Korona, Piotrków Trybunalski, Poland) with a 106 M UV Ozone Solution detector (Ozone Solution, Hull, MA, USA).

### 3.3. Determination of pH and Acidity

The total acidity (as citric acid) and the pH of the *L. kamtschatica* and *L. emphyllocalyx* fruits were analysed through the potentiometric titration of the sample for analysis with a standard 0.1 M NaOH solution at pH = 8.1 using TitroLine 5000 (SI Analytics, Weiheim, Germany) according to the method given in PN-EN 12147:2000 [42]. The results are expressed as g of citric acid per 100 g of fruit. Each fruit was analysed 24 h after ozonation. The analyses were performed in triplicate.

### 3.4. Determination of the Contents of Bioactive Compounds in Fruit and Determination of Their Antioxidant Activity

The ascorbic acid (Vitamin C) content was determined according to PN-A-04019:1998 [43]. The total polyphenol content (mg GAE·100 g^−1^ f.w.) was determined using the Folin–Ciocalteu method, according to the methodology described by Bakowska-Barczak et al., 2007 [44].

The antioxidant activity of the fruits was determined by using three different methods. DPPH antioxidant activity was measured according to a methodology given by Jurčaga et al., 2021 [45]; the results were expressed as the % of inhibition of DPPH radicals. The ABTS antioxidant activity was measured according to Gawroński et al., 2014 [46]; results were expressed in µM TE·g^−1^ f.w. The FRAP antioxidant activity was determined according to the methodology given by Rupasinghe et al., 2012 [7]; results are given in µM Fe^2+^·g^−1^ f.w. Each fruit was analysed 24 h after ozonation. All analyses were performed in triplicate.

### 3.5. Determination of the Morphological Characteristics of Berries

The sample size was 10 fruits from each variant. For individual fruits, the length and width [mm] were determined with an accuracy of 0.01 mm, and the weight was determined with an accuracy of 0.001 g. Each fruit was measured 24 h after ozonation.

### 3.6. Colour of L. kamtschatica and L. emphyllocalyx Berries

The colour analysis of the fruits was conducted via a reflection method by using a Chrome Meter colorimeter (Konica Minolta, Osaka, Japan) with a CR 400 head (Ø = 11 mm). The colorimeter was calibrated against a standard (No. 21833042) [47]. The reflectance method was applied at the standard lighting and 2° observer. The measurements results were read in the colorimetric system CIE LAB (CIE 1978)—L* (lightness), a* (redness), and b* (yellowness), taking three measurements for each sample. Each fruit was analysed 24 h after ozonation.

### 3.7. Statistical Analysis

Statistical analysis of the results was performed using Statistica 13.3. software (TIBCO Software Inc., Tulsa, OK, USA). The two-way analysis of variance (ANOVA) and LSD significance test were used with a significance level of α = 0.05. The Tukey test was used for statistical testing of the research results.

## 4. Conclusions

Based on this study, differences in fruit morphology and chemical composition were found between *L. kamtschatica* and *L*. *emphyllocalyx*. Use of gaseous ozone significantly increases the chemical composition of the tested varieties. The acidity of *L. emphyllocalyx* clones was, on average, 6% higher than the tested *L. kamtschatica* varieties. The *L. emphyllocalyx* clone ‘21-17’ had the highest average ascorbic acid content at 57.80 mg·100 g; the year of harvest significantly affected vitamin C levels, peaking at 53.92 mg·100 g in 2022. The total polyphenol content was also significantly influenced by the year of cultivation, with a 54.8% increase on average in 2022 compared to other years. The total antioxidant value, which was measured using the FRAP, DPPH, and ABTS methods, varied depending on the variety; ozone exposure significantly boosted antioxidant levels in all cases. On the basis of the study, there is significant improvement in the chemical composition of berries after exposure to gaseous ozone. The usage of ozone exposure was influential on the content of health-promoting compounds. The *L*. *emphyllocalyx* fruit differs significantly from its botanical variety, which may be useful in food processing. Further research on this botanical variety is required.

## Figures and Tables

**Table 2:** Morphological features and colour of *L. kamtschatica* and *L. emphyllocalyx* berries.

Variety	Length [mm]	Width [mm]	Weight [g]	L*	a*	b*
*L. caerulea* var. *kamtschatica*						
Vostorg	22.54 b	9.11 ab	1.21 b	27.74 a	3.82 c	−2.87 c
Jugana	23.49 b	9.28 ab	1.38 c	26.79 a	3.15 b	−2.43 b
Aurora	18.46 a	8.10 a	0.86 a	27.92 a	2.92 b	−2.45 b
*L. caerulea* var. *emphyllocalyx*						
Lori	19.30 a	8.95 a	1.49 c	27.94 a	2.45 a	−2.06 a
21-17	22.08 b	11.09 c	1.85 d	29.21 b	3.69 c	−2.49 b
139-24	18.90 a	12.07 c	2.01 e	27.76 a	2.88 b	−2.30 a
SL	***	***	***	*	***	***

Data are expressed as mean values (*n =* 3) ± SD; SD: standard deviation. Mean values with different letters are significantly different (*p* < 0.05). SL: level of significance; ns: not significant; ***: *p* < 0.001; * *p* < 0.05.

**Table 3:** The average monthly temperature and rainfall from 2022–2024 in the period of March to June in Tyczyn.

Year of Harvest	2022				2023				2024			
Month of Year	III	IV	V	VI	III	IV	V	VI	III	IV	V	VI
Average temperature [°C]	3	7	15	20	5.5	8.2	13.2	17.4	7	11	16	22
The lowest temperature in the month [°C]	−2.5	2.4	7.7	13	0.9	3.4	7.1	12	−3	1	5.7	10
The highest temperature in the month [°C]	8.9	12	21	27	7.7	13.3	18.9	23.1	9.5	17.2	20.5	23.1
Rainfall [mm]	28.1	51.1	38.8	27.3	79.8	45.9	52.1	45.4	23.1	35.6	59.4	67.3

**Table 4:** Harvest time of all analysed varieties in 2022–2024.

Variety	2022	2023	2024
Vostorg	15 May	17 May	5 May
Jugana	15 May	17 May	5 May
Aurora	25 May	29 May	17 May
21-17	12 June	12 June	5 June
139-24	6 June	8 June	26 May
Lori	6 June	8 June	30 May

## Data Availability

The original contributions presented in the study are included in the article, further inquiries can be directed to the corresponding author.
